# Adipose-derived stem cells prevent the onset of bisphosphonate-related osteonecrosis of the jaw through transforming growth factor β-1-mediated gingival wound healing

**DOI:** 10.1186/s13287-019-1277-y

**Published:** 2019-06-13

**Authors:** Xiaolong Zang, Linhai He, Lu Zhao, Yang He, E. Xiao, Yi Zhang

**Affiliations:** 1 Department of Oral and Maxillofacial Surgery, Peking University School and Hospital of Stomatology, 22 Zhongguancun Nandajie, Haidian District, Beijing, 100081 People’s Republic of China; 2 National Engineering Laboratory for Digital and Material Technology of Stomatology, Beijing Key Laboratory of Digital Stomatology, Beijing, China

**Keywords:** Bisphosphonate-related osteonecrosis of the jaw, Wound healing, Zoledronic acid, Fibroblast, Fibronectin, Adipose-derived mesenchymal stem cells

## Abstract

**Background:**

Due to its complex pathogenesis and low clinical cure rate, bisphosphonate-related osteonecrosis of the jaw (BRONJ) poses a substantial challenge for oral and maxillofacial surgeons. Therefore, the treatment of BRONJ should focus on prevention. In clinical studies, primary wound closure can significantly reduce the incidence of BRONJ. Whether local stem cell transplantation can promote primary gingival healing in patients with a medication history and prevent BRONJ has not been reported.

**Methods:**

In this study, animals were divided into a healthy group (non-drug treatment), a BP group, a hydroxyapatite (HA) group, and an adipose-derived stem cell (ADSC) group. All groups except the healthy group were treated with BPs and immunosuppressive drugs once per week for 8 weeks, simulating clinical use for the treatment of cancer patients with bone metastasis, to induce BRONJ-like animals. After the sixth drug treatment, the bilateral premolars were extracted in all groups. In contrast to the healthy and BP groups, the extraction sockets in the HA and ADSC groups were filled with HA or HA + ADSCs simultaneously post extraction to observe the preventive effect of ADSCs on the occurrence of BRONJ. At 2 and 8 weeks post extraction, animals from all groups were sacrificed.

**Results:**

At 8 weeks post transplantation, ADSCs prevented the occurrence of BRONJ, mainly through accelerating healing of the gingival epithelium at 2 weeks post extraction. We also found that ADSCs could upregulate the expression of transforming growth factor β1 (TGF-β1) and fibronectin in tissue from animals with a medication history by accelerating gingival healing of the extraction socket. A rescue assay further demonstrated that TGF-β1 and fibronectin expression decreased in TGF-β1-deficient ADSC-treated animals, which partially abolished the preventive effect of ADSCs on the onset of BRONJ.

**Conclusion:**

ADSCs prevent the onset of BRONJ, mainly by upregulating the expression of TGF-β1 and fibronectin to promote primary gingival healing, ultimately leading to bone regeneration in the tooth extraction socket. Our new findings provide a novel stem cell treatment for the prevention of BRONJ.

**Electronic supplementary material:**

The online version of this article (10.1186/s13287-019-1277-y) contains supplementary material, which is available to authorized users.

## Background

Bisphosphonate-related osteonecrosis of the jaw (BRONJ) is defined as a type of disease in which jaw bone is exposed in the maxillofacial region and persists for no less than 8 weeks in patients who are undergoing bisphosphonate treatment and have no oral radiation history [1]. BRONJ is a type of medication-related osteonecrosis of the jaw (MRONJ) and has been correlated with high doses of bisphosphonates (BPs) [2]. In addition, BRONJ was frequently observed in bone metastatic tumor patients who were treated in conjunction with immunosuppressive agents, and its incidence increased to 18% [3]. Dexamethasone, as the most common immunosuppressive drug, was used to treat multiple myeloma and bone metastasis and was also required for the development of a BRONJ-like disease model [4, 5]. The main clinical manifestations are impaired gingival wound healing and bone exposure, with necrotic bone formation accompanied by pain or sinus formation [1]. Among the risk factors, jaw trauma, especially tooth extraction and apical surgery, is a high-risk factor for BRONJ [6]. To date, this disease still lacks effective treatments, and only 59% of cases can be partially or completely healed [7]. In view of the low clinical cure rate, the treatment of BRONJ should focus on prevention.

Recent studies have indicated that primary closure after tooth extraction in BP-treated patients can effectively reduce the occurrence of BRONJ [8–10]. Notably, no MRONJ was observed when the tooth extraction wound was completely closed with a relaxation incision and/or sharp bone edges were removed, whereas an open wound increased the incidence to 56.1% [11]. Therefore, a soft tissue covering would reduce the occurrence of BRONJ.

Adipose-derived stem cells (ADSCs) are harvested from subcutaneous fat, are a rich source of cellular factors and promote angiogenesis, which makes them suitable for soft tissue repair [12–15]. ADSCs promote wound healing and antioxidation by paracrine cytokine activation [16, 17]. Stem cell-based treatment strategies are currently an effective method for the treatment of BRONJ. Notably, BRONJ-like lesions in mice and minipig models were successfully induced by BPs combined with dexamethasone and cured by systemic infusion of mesenchymal stem cells (MSCs) [5, 18]. Nevertheless, systemic infusion of MSCs may induce disseminated intravascular thrombosis and prolonged prothrombin time [19]. Most importantly, in cancer patients, systemic infusion of MSCs increased the risk of cancer metastasis and recurrence [20, 21]. To avoid the side effects mentioned above, in this study, we attempted to elucidate whether local application of ADSCs can also prevent the occurrence of BRONJ.

Overall, the present study evaluated the effect of local ADSC transplantation on tooth extraction in BP-treated animals to prevent the occurrence of BRONJ. We hypothesized that local ADSC transplantation would induce soft tissue repair and prevent the occurrence of BRONJ after tooth extraction.

## Methods

### Animals

New England rabbits (*n* = 36) weighing 2.0 kg were used in this study. The animals were housed in a conventional room with a regular dark-light cycle (12 h) at room temperature (20–25 °C). Experimental procedures involving animals followed institutionally approved protocols for animal research at Peking University (LA2018017).

### Reagents

Zoledronic acid was purchased from Sigma (SML0223, St. Louis, MO, USA); dexamethasone was purchased from Sinopharm Group (Shanghai Chemical Reagent Co. Ltd., China); the human TGF-β1 ELISA kit (DB-100B) and anti-TGF-β1 (MAB-240) primary antibody were purchased from R&D Systems (R&D Systems, Inc., Minneapolis, MN, USA); and the anti-fibronectin primary antibody was purchased from Santa Cruz (sc-59826, Santa Cruz Biotech, Santa Cruz, CA, USA).

### Induction of a BRONJ-like rabbit model and experimental design

Rabbits were maintained in a conventional room for 1 week to adapt to the environment. The BP, HA, and ADSC groups then received zoledronic acid (800 μg/kg, sml-0223, Sigma, USA) and dexamethasone (Dex, 10 mg/kg, Sinopharm Group, China) intravenously (I.V.) once per week for 8 weeks. The bilateral premolars were extracted under deep anesthesia by I.V. injection of pentobarbital (20 mg/kg, P3761, Sigma, USA) and xylazine (50 μl/kg, Jilin Huamu Animal Health Product Co., Ltd., China) after the sixth dose. For the ADSC group, ADSCs (passage 3–5) incubated on coral hydroxyapatite (HA) were immediately filled into the extraction sockets of animals with a medication history (ADSC group). Non-medication-treated animals and nonfilled animals (healthy group), medication-treated and nonfilled animals (BP group), and medication-treated and non-ADSC hydroxyapatite-filled animals (HA group) were used as controls. Then, at 2 and 8 weeks post extraction, the tooth extraction socket and the adjacent first molar were harvested at each indicated time. The unhealed area, necrotic bone, and new bone formation were measured based on clinical appearance and histology. All parameters mentioned above were quantified by ImageJ software (Version 1.51 s, US National Institutes of Health, Bethesda, Maryland).

### Formation of the ADSC-hydroxyapatite complex

The complex of ADSCs and hydroxyapatite was made based on the previously described method [22]. In brief, 5 × 10^6^ ADSCs in 300 μl of serum-free α-MEM medium per 40 mg of spheroidal hydroxyapatite/tricalcium phosphate (HA, Beijing YHJ Science and Trade Co. Ltd.) were used and preincubated at 37 °C for 30 min and then kept on ice before filling the socket.

### DiR fluorescence labeling of ADSCs

The commercially available lipophilic tracer 1,1-dioctadecyl-3,3,3,3-tetramethylindotricarbocyanine iodide (DiR; Molecular Probes Inc.) was used to label live ADSCs. In brief, 1 × 10^6^ ADSCs were stained with 1 ml (2.5 μg/ml) of DiR-labeling solution for 30 min at 37 °C, and the solution was then discarded after centrifugation (1500 rpm, 15 min) and resuspended in prewarmed (37 °C) phosphate-buffered saline (PBS). An extra centrifuge step was needed before incubation on HA.

### TGF-β1 ELISA

The concentrations of TGF-β1 in human gingival fibroblasts (HGFs) and ADSCs were measured with culture supernatant in serum-free medium with a TGF-β1 ELISA kit (R&D, USA) following the manufacturer’s instructions.

### Isolation and culture of ADSCs and HGFs

ADSCs were harvested from healthy donors. HGFs were harvested from healthy donors and BRONJ patients without bone metastases. All procedures were approved by the Ethics Committee of Peking University (IRB00001052-11002). The specific process is described in the Additional file 1: Supplemental methods.

### Flow cytometry

The ADSCs were washed 3 times with PBS and harvested by digestion and centrifugation. The cells were then incubated with the following labeled antibodies: CD90-FITC, CD44-PE, CD73-APC, CD105-CY5.5, and negative cocktail-PE. Control-conjugated immunoglobulin G (IgG) was used as the isotype control. All antibodies were purchased from BD Biosciences (No.562245). Flow cytometry was performed with an Accuri C6 flow cytometer (BD Biosciences).

### Multi-differentiation assays

For osteogenic differentiation, third-passage ADSCs were cultured in osteogenic medium supplemented with 10 nm dexamethasone, 0.1 mm l-ascorbic acid phosphate, and 10 mm β-glycerophosphate (Sigma-Aldrich). The osteogenic medium was changed every 3 days. After 7 days, messenger RNA (mRNA) was isolated for osteogenic genes analysis. After 21 days, calcium nodes were stained with 2% Alizarin red (Sigma-Aldrich).

For adipogenic differentiation, third-passage BMSCs were cultured in adipogenic culture medium supplemented with 0.5 μm hydrocortisone, 0.5 mm 3-isobutyl-methylxanthine, 10 μg/ mL insulin, 60 μm indomethacin (Sigma-Aldrich), and 10% FBS. After 7 days, mRNA was isolated for adipogenic genes analysis. After 21 days, the cells were stained for cellular lipid droplets with Oil Red O (Sigma-Aldrich).

### Immunochemistry-paraffin (IHC-P) staining

Immunochemistry was performed as previously reported [23]. In brief, the tissues were fixed in 4% formalin for 72 h at 4 °C, decalcified in 10% EDTA for 3 weeks until easily pierced by a needle and then embedded in paraffin for the next procedure. Slides (4 μm) were made and deparaffinized, and endogenous peroxidase was eliminated. After antigen blocking and retrieval, the slides were incubated with primary TGF-β1 and fibronectin antibodies overnight at 4 °C, thoroughly rinsed with PBS, incubated for 20 min and then stained with a DAB kit. The area of tissue with positive staining was quantified by ImageJ software (US National Institutes of Health).

### In vitro and in vivo siRNA interference assays

Double-stranded siRNA was used to silence TGF-β1 expression in ADSCs. The procedure and sequence used for the siRNA are available in the Additional file 1: Supplemental methods and Table S3. A satisfactory silencing efficiency in ADSCs was obtained after incubation on HA, and the cells were used in the animals with a medication history immediately post extraction (KD group).

### Tissue and cell RNA isolation and real-time polymerase chain reaction (real-time PCR)

To lyse tissues into a paste, a mortar with liquid nitrogen and a high-throughput tissue crusher (Qiagen GmbH, Hilden, Germany) were used for bone and gingival tissues. Both tissue and cell messenger RNA (mRNA) were isolated using TRIzol reagent (15,596, Invitrogen Corp., Carlsbad, CA). Messenger RNA was reverse transcribed into complementary DNA (cDNA) with the GoScript Reverse Transcription System (Promega, Madison, WI, USA), and an ABI Prism 7500 (Applied Bioscience, PerkinElmer, Foster City, CA) was used to perform quantitative PCR. The relative mRNA abundance of target genes was determined by normalizing to the GAPDH threshold cycle and calculated using the ^△△^Ct method. The primers are listed in Additional file 1: Tables S1 and S2.

### Western blot assay

Proteins were extracted from ADSCs and fibroblasts, and Western blot assays were performed as previously described [24]. Primary antibodies against GAPDH, transforming factor beta-1(TGF-β1), were purchased from R&D system (clone #9016).

### Bone parameter acquisition and analysis in different groups

The bone parameters were analyzed as previously described [25]. Computed tomography (CT) data were harvested by three-dimensional multi-image cone beam CT (60 kV, 2 mA, J. Morita Corp., Kyoto, Japan) and imported into the Inveon Research Workplace (SIEMENS, Munich, Germany) for further analysis. The region of interest was depicted, and the bone mineral density (BMD) and BV/TV were calculated.

### Tartrate-resistant acid phosphatase (TRAP) staining

TRAP staining was performed as previously described [26]. The specific process followed the instructions provided in the TRAP staining kit (Sigma-Aldrich, St. Louis, MO, USA), and the details are described in the Additional file 1: Supplemental methods.

### Statistical analysis

All data are presented as the mean ± STD from at least 3 independent animals. The differences between experimental groups were analyzed by using one-way analysis of variance (ANOVA) followed by Tukey’s honestly significant difference (HSD) post hoc test by using GraphPad Prism 6 (GraphPad Software). A difference was considered significant if *p* < 0.05 (indicated by an asterisk).

## Results

### Rabbit model of BRONJ-like lesions induced by BPs and DEX delivery

**Fig. 1:**
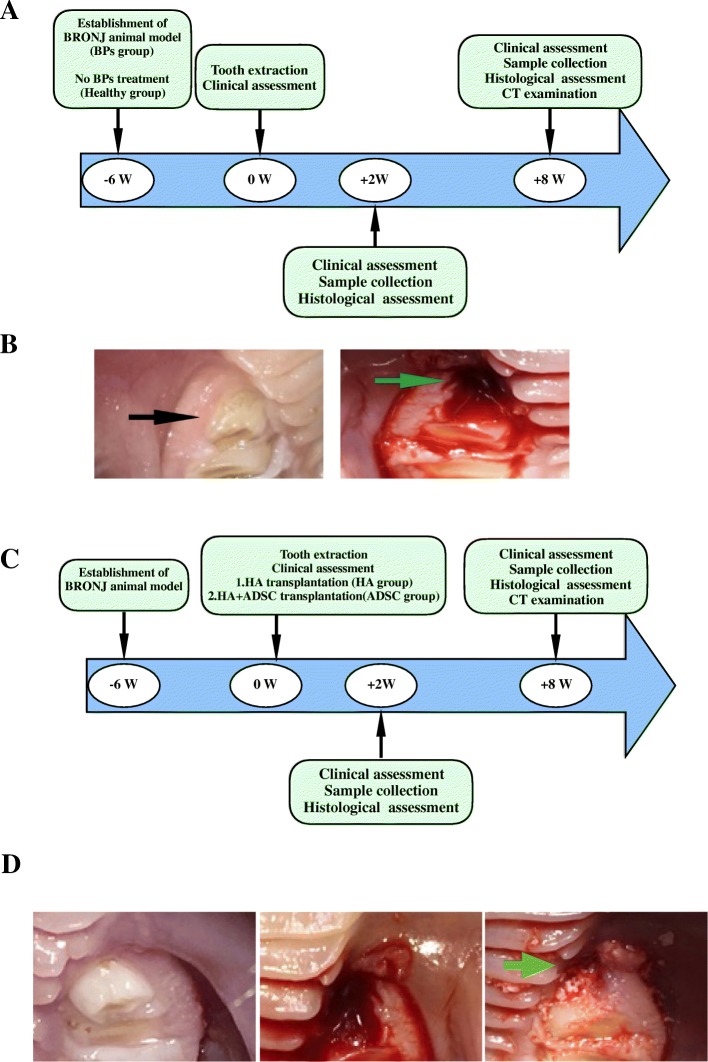
The treatment schedule used to induce BRONJ-like animals and the ADSC transplantation procedure. **a** Animals were divided into two groups, the healthy group (non-medication treated) and the BP group. The BP group was treated with zoledronic acid (ZOL) and dexamethasone (DEX). After the sixth dose of ZOL+DEX treatment, the bilateral premolars were extracted in both the healthy and BP groups. At 2 weeks and 8 weeks post extraction, samples were collected**. b** The procedure for tooth extraction and an extracted tooth (bar = 2 mm). **c** Animals were divided into two groups: the hydroxyapatite (HA) group and the ADSC group. Both groups were treated with ZOL+DEX once per week and followed the same schedule used to induce BRONJ-like animals. **d** The two groups were subjected to extraction of the bilateral premolars. Simultaneously, after tooth extraction, the HA group was filled with hydroxyapatite, and the ADSC group was filled with ADSCs that were incubated on HA. Arrows indicate filled HA or ADSC-HA complex

**Fig. 2:**
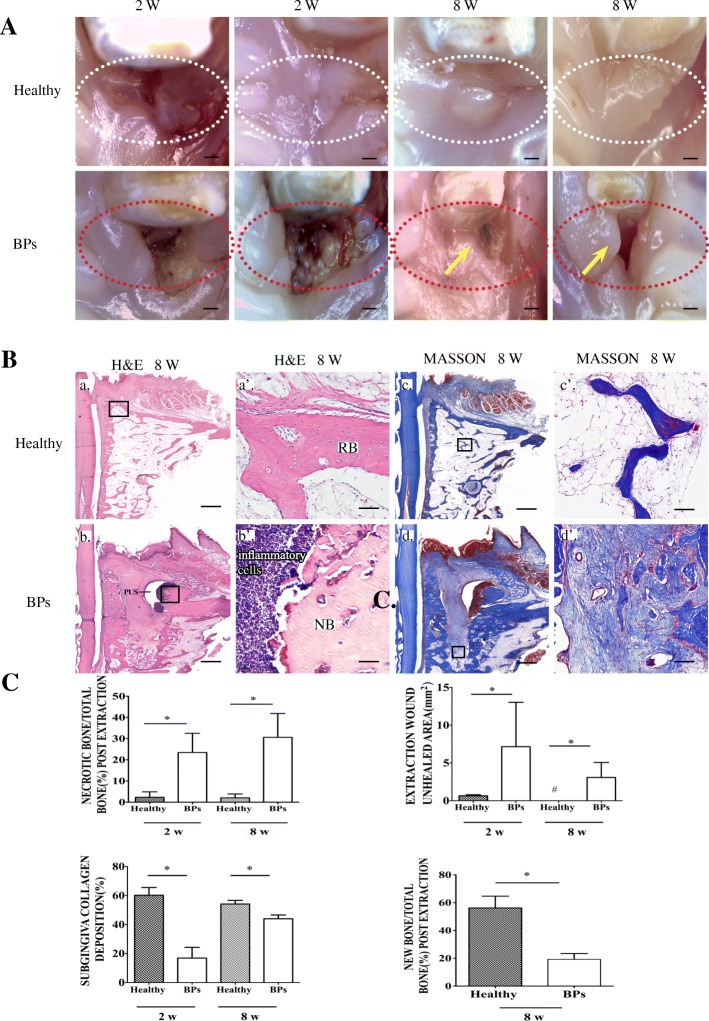
Induction of BRONJ-like animals by delivery of zoledronic acid and dexamethasone. **A** The clinical appearance of the healthy group and the BP group. The area in the dotted line shows the extracted tooth area at 2 and 8 weeks post extraction. Arrows indicate exposed jaw bone and unhealed gingiva (bar = 1 mm). **B** Hematoxylin and eosin (H&E) and trichrome staining (Masson) show bone regeneration and collagen deposition. Regenerated bone (RB). Necrotic bone (NB). The rectangular line indicates the area that was magnified and displayed on the side (a–d, bar = 1 mm; a’–d’, bar = 50 μm). **C** Quantitative analysis of the proportion of necrotic bone (%), subgingival collagen deposition (%) and unhealed area of the tooth extraction wound (mm^2^) at both 2 and 8 weeks post extraction and the proportion of new bone formation (%) at 8 weeks post extraction in the healthy and BRONJ groups. *p < 0.05, ^#^undetected data. The results are representative of three independent experiments

The induction of BRONJ-like animals followed the treatment schedule (Fig. 1a, b). At 8 weeks post extraction, the healthy group showed good healing (Fig. 2A) compared to the BP group, which exhibited retarded socket wound healing with partial jaw bone exposure. Histological analysis showed that extraction sockets from the healthy group presented a large proportion of cortical bone and cancellous bone (56.17 ± 8.51%) formation. However, in the BP group, cancellous bone formation was barely observed, with much less to the regenerated cortical bone (19.25 ± 4.16%) (Fig. 2B, C). Furthermore, necrotic bone was found in the BP group and was surrounded by bacterial infection, with pus formation. To investigate the collagen deposition in each group, Masson staining, which was quantified by using ImageJ, indicated that a greater proportion of collagen deposition beneath the gingival wound of the extraction socket was found in the healthy group (54.13 ± 2.53%) than in the BRONJ-like group (44.0 ± 2.61%) (Fig. 2B, C).

### Local transplantation of ADSCs promotes gingival wound healing and prevents the occurrence of BRONJ-like lesions

**Fig. 3:**
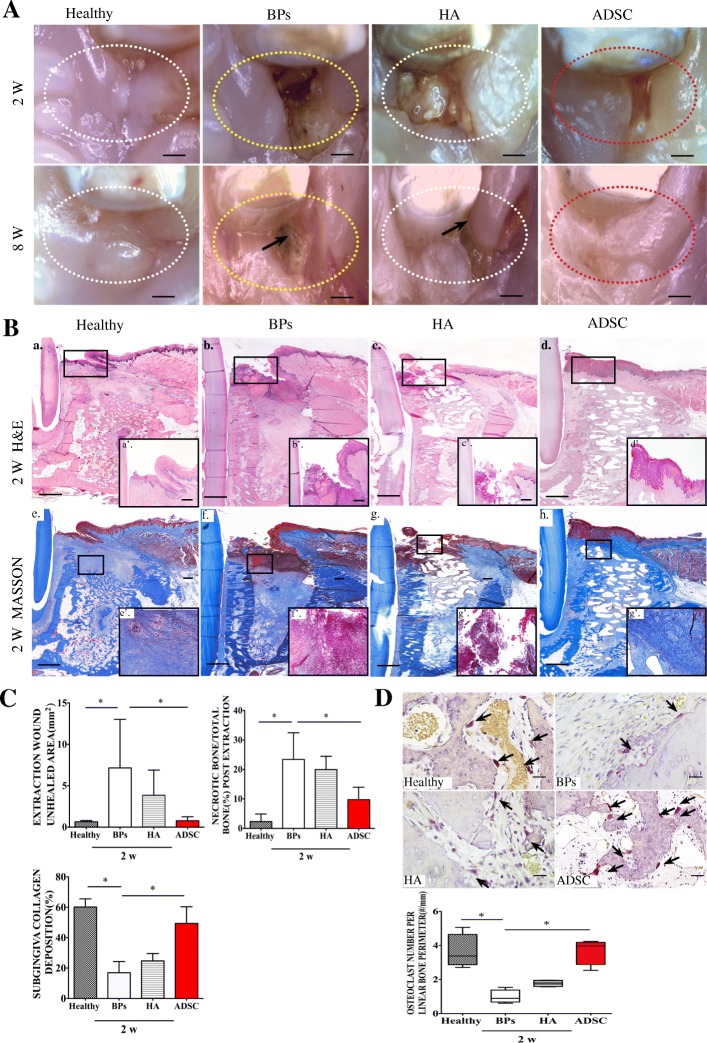
Local ADSC therapy promotes primary gingival healing at 2 weeks post extraction and facilitates bone regeneration at 8 weeks post extraction. **A** Clinical appearance of the healthy, BP, HA, and ADSC groups at 2 and 8 weeks post extraction. Areas in the dotted line show the tooth extraction site. Black arrows indicate jaw bone exposure in the BP group (bar = 1 mm). **B** H&E staining shows gingival healing in each group at 2 weeks post extraction. Masson staining indicates extraction site subgingival collagen deposition (a–h, bar = 1 mm; a’–d’, bar = 200 μm; e’–h’, bar = 50 μm). The rectangular line shows the magnified area. **C** Quantification of the unhealed area of the tooth extraction wound (mm^2^), the proportions of necrotic (%), and collagen deposition (%) in each group at 2 weeks. *p < 0.05. **D** TRAP-positive cells indicate osteoclasts. The arrows indicate osteoclasts, bar = 50 μm. Quantification of the number of osteoclasts per linear bone perimeter (#/mm), *p < 0.05

The cells, which we defined as adipose stem cells (ADSCs), were positive for CD44, CD90, CD105 and CD73 and were capable of osteogenesis and adipogenesis. (Additional file 1: Figure S1). Local transplantation of ADSCs was based on the described procedure (Fig. 1C, D). At 8 weeks after post extraction, both the ADSC and the healthy groups showed complete soft tissue coverage of the extraction socket, and no necrotic bone exposure was observed compared with the BP and the HA groups (Fig. 3A). Notably, local transplantation of ADSCs induced primary gingival wound healing at 2 weeks post extraction, which indicated that the unhealed extraction wound area (mm^2^) was smaller after local transplantation of ADSCs (0.77 ± 0.48 mm^2^), and this outcome was more consistent with the healthy group (0.647 ± 0.144 mm^2^) than the BP (7.152 ± 5.874 mm^2^) and HA (3.86 ± 3.03 mm^2^) groups (Fig. 3A, C). Interestingly, with the early gingival covering at 2 weeks post extraction, less inflammatory infiltration was found under the socket connective tissues after local transplantation of ADSCs (Fig. 3B). The proportion of collagen deposition in the subgingival connective tissue of the tooth extraction socket was significantly higher in the ADSC and healthy groups than in the BP and HA groups 2 weeks after extraction (49.36 ± 11% and 60.18 ± 5.35% vs. 16.90 ± 7.37% and 23.35 ± 4.96%), suggesting that ADSCs promote subgingival connective tissue collagen deposition and accelerate the healing of gingival wounds (Fig. 3C). Local ADSC transplantation also rescued the number of osteoclasts per linear bone perimeter (#/mm) compared to the BP group (Fig. 3D), indicating a revived bone remodeling process. Thus, local ADSC transplantation can promote gingival wound healing and collagen deposition.

### Primary gingival healing by transplantation of ADSCs facilitate socket bone regeneration and upregulate osteogenic genes expression

**Fig. 4:**
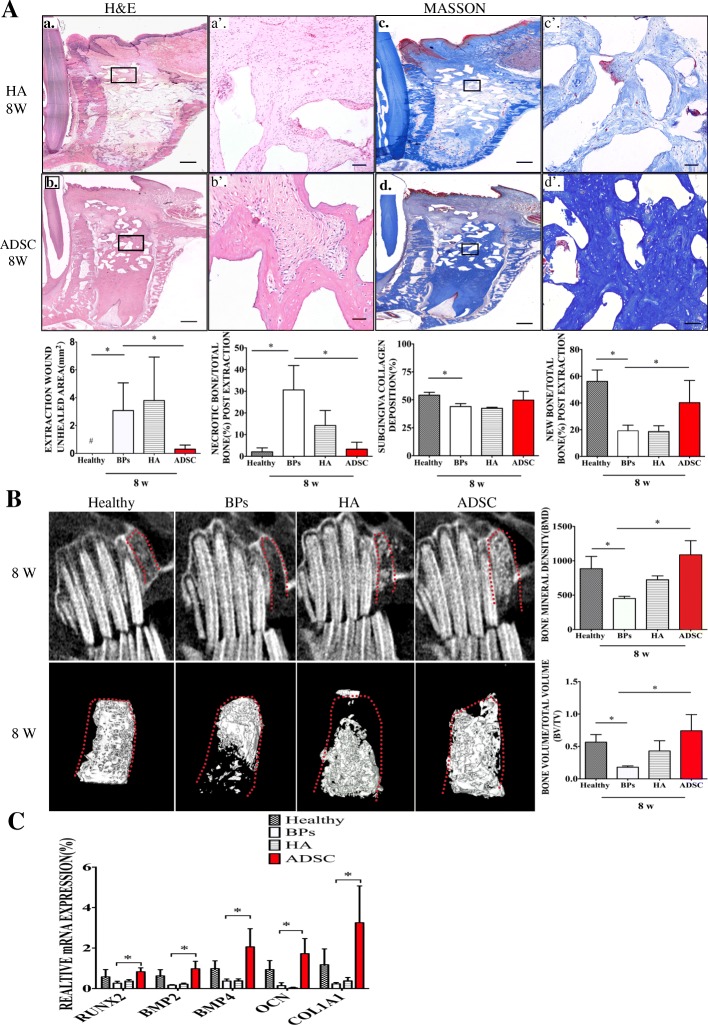
Primary gingival healing by transplantation of ADSCs facilitate socket bone regeneration and upregulate osteogenic genes expression. **A** Local ADSC therapy facilitates socket bone regeneration. H&E and Masson staining indicate bone regeneration in the ADSC and HA groups (a–d, bar = 1 mm; a’–d’, bar = 50 μm). Quantification of the unhealed area of the tooth extraction wound (mm^2^), the proportion of necrotic bone (%), new bone formation (%), and extraction socket subgingival collagen deposition (%) in each group at 2 weeks post extraction. *p < 0.05, ^#^undetected data. **B** Computed tomography (CT) examination shows reconstructed three-dimensional (3D) images and quantification of bone volume/total volume (BV/TV) and bone mineral density (BMD) in each group. *p < 0.05. The results are representative of three independent experiments. **C** Relative expression of osteogenic target genes in each group. *p < 0.05

Following the primary gingival wound healing observed at 2 weeks post extraction, a large proportion of new bone regeneration (40.23 ± 16.62%) was also found in the ADSC group at 8 weeks post extraction (Fig. 4A). Histological analysis (H&E and Masson staining) indicated a lower proportion of necrotic bone in the ADSC group (3.30 ± 3.18%), which showed a pattern similar to that in the healthy group (2.075 ± 1.81%), rather than that in the BP (30.60 ± 11.24%) and HA groups (14.21 ± 6.94%) (Fig. 4A). Cone beam computed tomography (CBCT) examination revealed that the extraction sockets in the ADSC and healthy groups were mostly occupied by regenerative bone, but no obvious new bone regeneration was observed in the BP and HA groups (Fig. 4B), which is consistent with the osteogenic genes relative expression in bone tissue being significantly upregulated in the ADSC and healthy groups than in the BP group (Fig. 4C). Bone volume/total volume (BV/TV) and BMD in the ADSC group were significantly increased compared with those in the BP and HA groups and similar to those in the healthy group (Fig. 4B).

### Local ADSC transplantation improved suppressed TGF-β1 and fibronectin expression in BRONJ-like rabbits

**Fig. 5:**
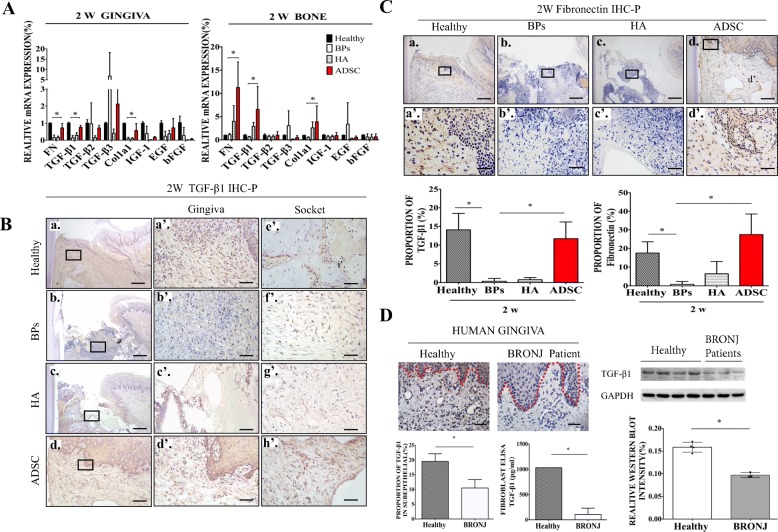
Local ADSC transplantation improved suppressed TGF-β1 and fibronectin expression in BRONJ-like rabbits. **A** Assessment of the target genes in each group. Relative tissue messenger RNA (mRNA) expression levels of target genes in each group at 2 weeks post extraction. *p < 0.05. Immunohistochemical detection of TGF-β1 (**B**) and fibronectin (FN) (**C**) abundance and quantification of their expression in the subgingiva of the extraction socket in each group. *p < 0.05; **B** a–d, bar = 200 μm; a’–h’, bar = 20 μm; **C** a–d, bar = 200 μm; a’–d’, bar = 20 μm. The rectangular line shows the magnified area. **D** Immunohistochemistry showing TGF-β1 expression in gingival tissue from a BRONJ patient and quantified expression. An ELISA kit and western blot were used to detect TGF-β1 expression in gingival fibroblasts (HGFs) from three BRONJ patients. *p < 0.05, bar = 20 μm

According to previous results, the gingival epithelium was mostly healed in the ADSC and healthy groups but not in the BP and HA groups at 2 weeks post extraction, which indicated that ADSCs promote gingival wound healing within 2 weeks post extraction (Fig. 3A). DiR was used as a fluorescent stain to label live ADSCs, which verified that ADSCs survived for 2 weeks after tooth extraction (Additional file 1: Figure S2). To further investigate the mechanism by which ADSCs promote primary gingival wound healing, total gingiva and bone tissue were harvested, and mRNA levels were determined by quantitative PCR. TGF-β1 and fibronectin were the most significantly downregulated in the BP and HA groups compared with the healthy group; however, both of these factors were highly expressed in the ADSC group (Fig. 5A). The same pattern was further confirmed by immunohistochemistry, which showed a higher expression of TGF-β1 and fibronectin in the ADSC group than that in the BP and HA groups (Fig. 5B, C). Furthermore, patients’ gingival samples from BRONJ lesions confirmed impaired TGF-β1 expression in BP gingival tissue. The TGF-β1 levels of patients’ gingival fibroblasts were further verified a decreased pattern by ELISA assay and western blot (Fig. 5D). Thus, ADSCs may prevent the onset of BRONJ by inducing TGF-β1 and fibronectin expression.

### TGF-β1 deficiency eliminated the ability of ADSCs to accelerate gingival wound healing in BP-treated animals

**Fig. 6:**
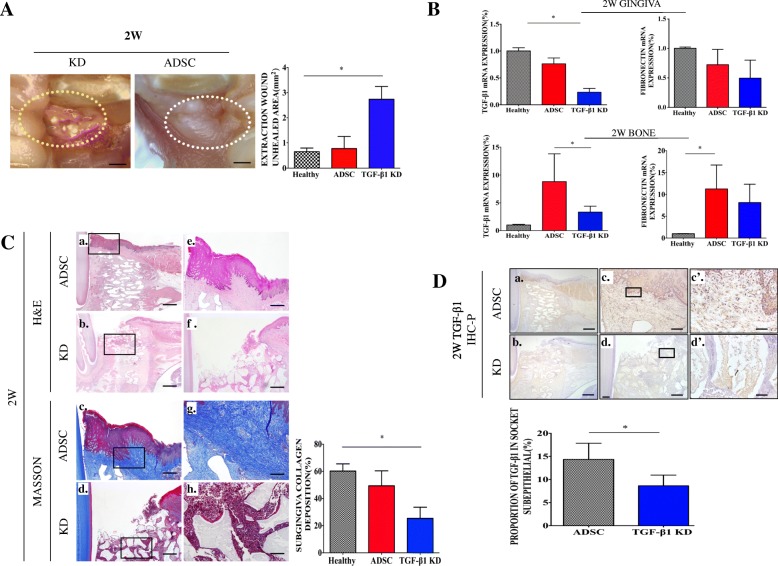
TGF-β1 played a pivotal role in ADSC-mediated gingival healing. **A** Clinical manifestations at the extraction site in the ADSC group and KD group. The area in the dotted line indicates the tooth extraction site. **B** TGF-β1 and FN relative expression in gingival and bone tissue from the KD group compared to that in the ADSC and healthy groups. *p < 0.05. **C** H&E and Masson staining show gingival healing. Unhealed area of the tooth extraction wound (mm^2^) and extraction socket subgingival collagen deposition (%) were quantified. *p < 0.05; a–b, bar = 1 mm; c–f, bar = 200 μm; g–h, bar = 50 μm. Immunohistochemistry to detect TGF-β1 (**D**) expression in the KD group and ADSC group and quantification of their expression in the subgingiva of the extraction socket. *p < 0.05. **D** a–b, bar = 1 mm; c–d, bar = 200 μm; c’–d’, bar = 20 μm. The rectangular line shows the magnified area. The results are representative of three independent experiments

TGF-β1 played a pivotal role in ADSC-mediated acceleration of wound healing [27–29]. Moreover, the expression of TGF-β1 follows positive feedback regulation [30]. To test the role of TGF-β1 in ADSC-mediated accelerated gingival wound healing in BP-treated animals, siRNA was applied to knock down TGF-β1 expression in ADSCs during BRONJ-like prevention, as described above. The silencing efficiency of TGF-β1 was confirmed by real-time PCR, ELISA, and western blotting (Additional file 1: Figure S3). The unhealed extraction wound area in the KD group (2.20 ± 0.35 mm^2^) was larger than that in the ADSC group (0.77 ± 0.48 mm^2^) (Fig. 6A). Real-time PCR indicated that both TGF-β1 and fibronectin were suppressed in the KD group (Fig. 6B). Histological analysis revealed histological features similar to those of the BP group, which exhibited partial inflammatory infiltration beneath the gingival tissue and less collagen deposition (25.42 ± 8.22%) in connective tissue than the ADSC group (49.36 ± 11%) (Fig. 6C). Immunohistochemistry showed that TGF-β1 expression in the subgingiva was significantly diminished in the KD group compared to that in the ADSC group (Fig. 6D), which verified that TGF-β1 played a pivotal role in ADSC-accelerated gingival healing.

## Discussion

The highest incidence of BRONJ is observed in malignant tumor patients treated with a combination of BPs and DEX. However, based on the current treatment method, the cure rate of BRONJ is only 58.5% [31]. It is particularly important to prevent the occurrence of BRONJ in patients with a medication history following a tooth extraction or other dentoalveolar surgery. In our study, we successfully induced a BRONJ-like rabbit model by simulating the clinical application of BPs and immunosuppressive drugs. BRONJ was successfully prevented when ADSCs were transplanted into the extraction wound. We first showed that ADSC transplantation elevated TGF-β1 and fibronectin expression under BRONJ conditions, contributing to the promotion of gingival wound healing and facilitating bone regeneration. Furthermore, when we knocked down TGF-β1 in ADSCs, these cells failed to promote primary gingival wound healing. In summary, local ADSC transplantation proved that ADSCs can mediate gingival wound healing through TGF-β1, and early soft tissue healing accelerated by ADSCs is important for preventing the onset of BRONJ.

In our study, we found that TGF-β1 and fibronectin expression was suppressed not only in BP-treated animal but also in patient gingival lesions, which may be connected to delayed gingival healing in BRONJ. During normal wound healing, fibronectin can induce the regeneration of epithelial tissue by recognizing the RGD sequence on the epithelial cell integrin receptor α5β1 [32, 33]. However, the absence of fibronectin expression in irradiated skin leads to skin wounds that do not heal properly [34]. It was also reported that fibronectin is regulated by TGF-β1 in a c-Jun N-terminal kinase-dependent and Smad4-independent manner [35]. Other studies also showed that BPs could inhibit the TGF-β1 pathway [36], leading to collagen deposition and restricted downstream signaling. The retarded gingival healing in BRONJ may be caused by suppressed fibronectin regulated by low levels of TGF-β1.

Retarded gingival healing with jaw bone exposure is the key clinical manifestation of BRONJ. Many studies have proven that soft tissue covering is the key factor in preventing BRONJ by mitigating secondary contamination and re-establishing a vascularized tissue bed that may nourish the diseased bone tissue [8, 37, 38]. Soft tissue is superior in terms of promoting inferior bone regeneration, as it prevents wound infection, serves as a local source of growth factors, and upregulates bone remodeling [39–42]. Soft tissue also provides a bone anabolic environment through the expression of members of the TGF-β superfamily of growth and differentiation factors, including the bone morphogenetic proteins (BMPs), osteocalcin (OCN), and receptor activator of nuclear factor kappa-B ligand (RANKL) [42–44]. However, delayed gingival healing led to the invasion of bacteria into the tooth extraction wound, contributing to inflammatory infiltration and the development of BRONJ [37]. In our study, local transplantation of ADSCs accelerated gingival healing and formed a sealed internal environment. We also found that early gingival healing significantly reduced inflammatory infiltration in subgingival connective tissue and provided a stable internal environment for bone regeneration. In addition, with coverage of the gingival tissue, we found that activated osteoclasts were rescued and that the expression of BMP-2, OCN, and other osteogenesis-related genes in the bone tissue was elevated. Therefore, the prevention of BRONJ by ADSCs is mainly achieved by the ability of the cells to promote early gingival healing and isolate the wound environment from the oral cavity, thus facilitating bone remodeling.

An increasing number of studies have proved that secreted cytokines perform the major therapeutic functions of transplanted stem cells [45, 46]. TGF-β1 is an important cytokine, particularly in ADSC-mediated wound healing. This cytokine can promote epithelial healing by mediating extracellular matrix (ECM) synthesis through upregulation of fibroblast collagen type I and III and fibronectin expression and facilitating fibroblast migration and proliferation to promote granulation tissue formation and reepithelization [27, 47, 48]. Even under hypoxic conditions, stem cells enhance TGF-β1 expression, accelerating dermal fibroblast migration, further elevating fibronectin expression and ultimately, promoting skin wound healing [49]. In addition, due to low expression of TGF-β1 in diabetic ulcers, ADSCs can promote epithelial regeneration and granulation tissue formation by supplementing exogenous TGF-β1, thus promoting wound healing [50, 51]. Consistent with these studies, we also found that impaired gingival healing is accompanied by less TGF-β1 expression in BRONJ. In addition, local ADSC transplantation increased TGF-β1 expression and accelerated gingival healing. To further clarify the role of TGF-β1 in ADSC-mediated gingival healing, we knocked down TGF-β1, which eliminated the healing effects of ADSCs in BP-treated animals. Therefore, we believe that ADSCs induce gingival healing mainly mediated by TGF-β1 and thus prevent the onset of BRONJ.

In summary, we discovered that local ADSC transplantation provides a safe and effective therapeutic modality for preventing the onset of BRONJ by promoting primary gingival healing and represents a promising approach to clinical stem cell treatment.

## Conclusions

We have revealed that local transplantation of ADSCs in animals with a medication history could prevent the onset of BRONJ, mainly by upregulating TGF-β1 and fibronectin expression and promoting gingival healing. Furthermore, TGF-β1 secreted from ADSCs is essential in the effects of primary gingival wound healing. This study provides a new perspective concerning how local stem cells act to prevent the occurrence of BRONJ.

## Additional file

Additional file 1: Detailed methodology and supplementary data. **Figure S1**. Identification and multi-differentiation of Adipose-derived stem cells(ADSCs). **Figure S2**. ADSCs labeled with DiR were shown in the tooth extraction area. **Figure S3**. TGF-β1 gene silencing efficiency detected by mRNA expression and protein level. **Table S1**. Rabbit primers Used in Real-time Polymerase Chain Reaction Gene Expression Analysis. **Table S2**. Human primers Used in Real-time Polymerase Chain Reaction Gene Expression Analysis. **Table S3**. The double-stranded sequence of siRNA from RiboBio company. (DOCX 17673 kb)

## Data Availability

All data generated or analyzed during this study are included in this published article. All methods and materials used in this study are listed in Additional file 1.

## Abbreviations

ADSCs: Adipose-derived stem cells
ALP: Alkaline phosphatase
bFGF: Basic fibroblast growth factor
BMP: Bone morphogenic protein
BPs: Bisphosphonates
BRONJ: Bisphosphonate-related osteonecrosis of the jaw
cDNA: Complimentary DNA
Col1a1: Collagen type 1a1
DEX: Dexamethasone
EGF: Epidermal growth factor
FN: Fibronectin
HA: Hydroxyapatite
IGF: Insulin-like growth factor
LPL: Lipoprotein lipase
mRNA: Messenger RNA
MRONJ: Medication-related osteonecrosis of the jaw
MSCs: Mesenchymal stem cells
OCN: Osteocalcin
PPAR-γ: Peroxisome proliferator-activated receptors
RUNX-2: Runt-related transcription factor 2
TGF: Transforming growth factor
