# Supplementary material for: Adipose-derived stem cells prevent the onset of bisphosphonate-related osteonecrosis of the jaw through transforming growth factor β-1-mediated gingival wound healing
Source: Stem Cell Res Ther. 2019 Jun 13;10:169. doi: 10.1186/s13287-019-1277-y (PMC6567445; doi:10.1186/s13287-019-1277-y)
Supplement: Supplementary file 1 — Detailed methodology and supplementary data. Figure S1. Identification and multi-differentiation of Adipose-derived stem cells(ADSCs). Figure S2. ADSCs labeled with DiR were shown in the tooth extraction area. Figure S3. TGF-β1 gene silencing efficiency detected by mRNA expression and protein level. Table S1. Rabbit primers Used in Real-time Polymerase Chain Reaction Gene Expression Analysis. Table S2. Human primers Used in Real-time Polymerase Chain Reaction Gene Expression Analysis. Table S3. The double-stranded sequence of siRNA from RiboBio company. (DOCX 17673 kb) [file 13287_2019_1277_MOESM1_ESM.docx]

**Additional File 1 Content**

| **Order** | **Contents** |
| --- | --- |
| 1 | Supplementary Materials and Methods |
| 2 | Supplemental Figures |
| 3 | Tables |

1. **Supplementary Materials and Methods**

**Isolation and culture of ADSCs and human gingival fibroblasts (HGFs)**

Tissues were thoroughly rinsed with sterile phosphate-buffered saline (PBS), cut into a tissue paste by using a lancet and digested in a solution containing dispase II (4 mg/mL, 494207800, Roche, Indianapolis, USA) and collagenase I (2 mg/mL, C0130-1G, Sigma, USA) at 37°C for 20 min. After digestion, the suspension was centrifuged (1200 rpm, 5 min) and resuspended. The final suspension was passed through a 70-μm filter (BD Biosciences, San Jose, CA), and the flow-through and lysed tissues were collected and seeded in culture medium consisting of α-modified Eagle medium (α-MEM, C12571500BT, Gibco, Grand Island, NY, USA) supplemented with 10% fetal bovine serum (FBS; Equitech-Bio, Inc., Kerrville, TX, USA), penicillin G and streptomycin sulfate (15240062, Gibco, USA) at 37°C in a humidified atmosphere with 5% CO2 for 24 h. After 24 h, the medium was exchanged with fresh medium (α-MEM with 10% FBS and penicillin G and streptomycin sulfate) and continuously cultured.

**Tartrate-resistant acid phosphatase (TRAP) staining**

Tissues were obtained from each group to form tissue slides (4 μm) as described in the IHC-P staining method. Four slides were randomly selected from each group to perform TRAP (Sigma-Aldrich, St. Louis, MO, USA) staining according to the manufacturer’s instructions. The cells stained purplish-red with at least three cell nuclei were obtained. The number of osteoclasts in the tooth extraction area of each group was calculated and averaged by the total perimeter of the tooth extraction area via a BIOQUANT OSTEO Bone Biology Research System (BIOQUANT Image Analysis Corporation, Nashville, TN, USA). The average number of osteoclasts was calculated by the total number of osteoclasts in the tooth extraction area divided by the total tooth extraction area perimeter. The osteoclast number per tooth extraction area (i.e., per circumference of bone marrow in millimeters) was presented for each group.

**In vitro siRNA interference procedures**

In brief, ADSCs (passage 3-5) were plated at a concentration of 150,000 cells/well in six-well plates in complete medium (α-MEM supplemented with 10% FBS, penicillin G and streptomycin sulfate). The transfection medium was made with opti-MEM (Invitrogen, Carlsbad, CA, USA) combined with TGF-β1-specific or scrambled siRNA (RiboBio Co. Ltd., Guangzhou, Guangdong) and Lipofectamine RNAi-max (Invitrogen, Carlsbad, CA, USA) according to the instructions. ADSCs were transfected for 48 h, after which the medium was replaced with siRNA-free α-MEM for 3 days. ADSCs were homogenized in lysis buffer for RNA extraction, and supernatants were also collected for ELISA.

1. **Supplemental figures**

**Figure S1**

**Figure S1.** **Identification and muti-differentiation of adipose-derived stem cells(ADSCs).** (A) ADSCs identification by flow cytometry analysis. The differentiation potential of ADSCs stained with (B) alizarin red S or (C) Oil red O (scale bar = 20 μm) is shown; (D) Realtime-PCR showed that adipogenic genes, PPAR-γ and LPL, and osteogenic genes, ALP, OCN and Runx-2, were higher expressed than control ADCSs.

**Figure S2**

**
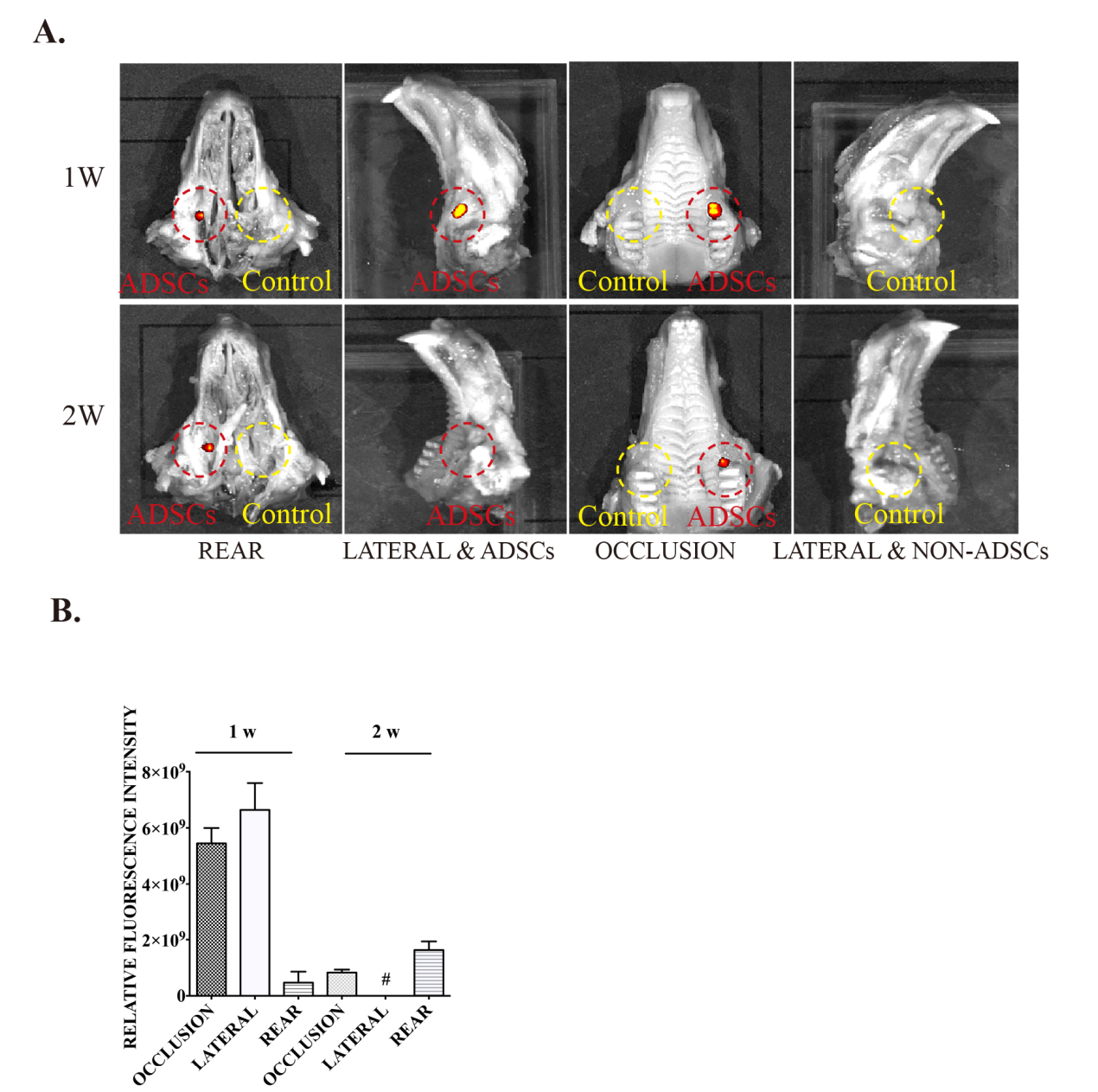
**

**Figure S2. ADSCs labeled with DiR were shown in the tooth extraction area.** DiR was used as a fluorescent stain to label live ADSCs, which verified that ADSCs survived for no less than 2 weeks after tooth extraction, as visualized by an IVIS Lumina III system (PerkinElmer, Inc., Spokane, WA, USA). (A) The area in the red dotted line indicates fluorescently labeled ADSCs. The area in the yellow dotted line indicates non-fluorescently labeled ADSCs that served as controls. (B) Quantification of the fluorescence intensity, # undetected data.

**Figure S3**

**Figure S3. TGF-β1 gene silencing efficiency detected by mRNA expression and protein level.** TGF-β1 gene silencing efficiency detected by mRNA expression, ELISA kit(A) and western blot (B).

1. **Tables**

**Table S1. Rabbit primers Used in Real-time Polymerase Chain Reaction Gene Expression Analysis.**

| Gene | Primier sequence | | | |
| --- | --- | --- | --- | --- |
| GAPDH | Forward  Reverse | AGACACGATGGTGAAGGTCG  TGCCGTGGGTGGAATCATAC |  |  |
| TGF-β1  TGF-β2 | Forward  Reverse  Forward  Reverse | TGTCACTGGAGTTGTGAGGC  AGCAGTTCTTCTCTGTGGAGC  ACACTTTGGAAGTTTGTGTTCTGTT  GGGAAGGGTGCCTATTGCAT |  |  |
| TGF-β3 | Forward  Reverse | GGGCTGCTCGGAGTAACTTT  GGAAAAGCCGAGCCCATTTG |  |  |
| COL1A1  Fibronectin  IGF-1  bFGF  BMP-2  BMP-4  OCN  RunX-2  ALP | Forward  Reverse  Forward  Reverse  Forward  Reverse  Forward  Reverse  Forward  Reverse  Forward  Reverse  Forward  Reverse  Forward Reverse  Forward Reverse | CGATGGCTTCCAGTTCGAGT  TCGTGGAGGACAGTGTAGGT  CTCACCCGAGGCGCCACCTA  TCGCTCCCACTCCTCTCCAACG  TGTGATCTGAGGAGGCTGGA  ACTTGTGTTCTTCAAATGTACTTCC  AGCGGCTGTACTGCAAAAA  TGCACACACACCTTTGATGG  ACCACCCGGCGATTCTTCTTT  AACCGCTGTCGTCTCCCAAA  CCACCACGAAGAACATCTGGA TGTTTATCCGGTGGAAGCCC  CTCACACTCCTCGCCCTATT  CGCCTGGGTCTCTTCACTAC  AGTTTGTTCTCTGACCGCCTC  GCTGGGCTCTGAATCTGAAATG  AGTTTGTTCTCTGACCGCCTC  GCTGGGCTCTGAATCTGAAATG |  |  |

**Table S2. Human primers Used in Real-time Polymerase Chain Reaction Gene Expression Analysis.**

| Gene | Primier sequence | |
| --- | --- | --- |
| Actin-β  TGF-β1  PPAR-γ  LPL  OCN  ALP  Runx-2 | Forward  Reverse  Forward  Reverse  Forward  Reverse  Forward  Reverse  Forward  Reverse  Forward  Reverse  Forward  Reverse | CATGTACGTTGCTATCCAGGC  CTCCTTAATGTCACGCACGAT  CTAATGGTGGAAACCCACAACG  TATCGCCAGGAATTGTTGCTG  GGGATCAGCTCCGTGGATCT  TGCACTTTGGTACTCTTGAAGTT  TCATTCCCGGAGTAGCAGAGT  GGCCACAAGTTTTGGCACC  CACTCCTCGCCCTATTGGC  CCCTCCTGCTTGGACACAAAG  AACATCAGGGACATTGACGTG  GTATCTCGGTTTGAAGCTCTTCC  TGGTTACTGTCATGGCGGGTA  TCTCAGATCGTTGAACCTTGCTA |

**Table S3. The double-stranded sequence of siRNA from RiboBio company.**

| Series Number | Sequence |
| --- | --- |
| siG091112132209 | GCAGAGTACACACAGCATA |
